# Disentangling Multispectral Functional Connectivity With Wavelets

**DOI:** 10.3389/fnins.2018.00812

**Published:** 2018-11-06

**Authors:** Jacob C. W. Billings, Garth J. Thompson, Wen-Ju Pan, Matthew E. Magnuson, Alessio Medda, Shella Keilholz

**Affiliations:** ^1^Graduate Division of Biological and Biomedical Sciences – Program in Neuroscience, Emory University, Atlanta, GA, United States; ^2^Biomedical Engineering, Georgia Institute of Technology and Emory University, Atlanta, GA, United States; ^3^iHuman Institute, ShanghaiTech University, Pudong, China; ^4^Aerospace Transportation and Advanced Systems, Georgia Tech Research Institute, Atlanta, GA, United States

**Keywords:** resting state, functional magnetic resonance imaging, functional connectivity, wavelet packet transform, mutual information, clustering

## Abstract

The field of brain connectomics develops our understanding of the brain's intrinsic organization by characterizing trends in spontaneous brain activity. Linear correlations in spontaneous blood-oxygen level dependent functional magnetic resonance imaging (BOLD-fMRI) fluctuations are often used as measures of functional connectivity (FC), that is, as a quantity describing how similarly two brain regions behave over time. Given the natural spectral scaling of BOLD-fMRI signals, it may be useful to represent BOLD-fMRI as multiple processes occurring over multiple scales. The wavelet domain presents a transform space well suited to the examination of multiscale systems as the wavelet basis set is constructed from a self-similar rescaling of a time and frequency delimited kernel. In the present study, we utilize wavelet transforms to examine fluctuations in whole-brain BOLD-fMRI connectivity as a function of wavelet spectral scale in a sample (*N* = 31) of resting healthy human volunteers. Information theoretic criteria measure relatedness between spectrally-delimited FC graphs. Voxelwise comparisons of between-spectra graph structures illustrate the development of preferential functional networks across spectral bands.

## Introduction

The advent of functional magnetic resonance imaging (fMRI) offers an unprecedented view into normal brain function (Ogawa et al., 1990; Bandettini, 2012). One of the earliest uses of fMRI was to localize areas of the brain involved in experimentally defined tasks. Changes in blood-oxygen level dependent (BOLD) signals were statistically compared between task and control states (Belliveau et al., 1991). However, these task-related activations account for relatively small deviations (5–10%) from baseline metabolism (Raichle and Mintun, 2006). Biswal et al. (1995) analyzed the structure of the BOLD signal's spontaneous fluctuations to discover that temporal correlations in the low-frequency BOLD signal demarcate the same regions of the brain as activated during certain tasks. Mapping networks of “functional connectivity” (FC) based on intrinsic BOLD correlations has since become a powerful tool for neuroscience research. Among normal adults, contiguous brain networks (visual network, somatomotor network, cerebellar network, etc.) and networks composed of multiple disconnected regions (the default mode network, the dorsal attention network, etc.) are non-invasively identified through FC-fMRI (Fox et al., 2005; Vincent et al., 2008; Smith et al., 2009; Yeo et al., 2011).

Spontaneous BOLD fluctuations have been shown to match a 1/f-type scaling of frequency, *f*, to power spectral density, *S*: *S*(*f*) ∝ 1/*f*^γ^ (He, 2014). The spectral exponent, γ, has a value of between 0.5 and 1 in BOLD data (Bullmore et al., 2004; Herman et al., 2011). The physiological significance of 1/f-type scaling of brain signals is hotly debated. Conceptually, natural 1/f-type systems emerge as large-scale realizations of many granular and self-similar details. For instance, the 1/f-distributed BOLD signal has been demonstrated to be a convolution of discrete neural signaling events with a hemodynamic response function (Logothetis et al., 2001). Some authors discount multispectral features from 1/f-type signals as “scale-free” organization—that is, the 1/f-type scaling indicates that a finite set of properties describes the systems structures at all scales (Goldberger et al., 2002; Mandelbrot, 2013). Other authors point to fluctuations in the spectral exponent across brain regions and between task and rest conditions as an indication that variance in the multispectral evolution of brain signals bears useful information (He et al., 2008; He, 2011, 2014). The fact of the BOLD signal's mean and deviant 1/f-type structure motivates domain transformation that model spectral variability (Medda et al., 2016; Bielczyk et al., 2017; Billings, 2017; Shakil et al., 2017).

Perfectly scale-free systems may be constructed via tessellations of self-similar fractals. Wavelet transforms offer theoretically optimal domains for investigating 1/f-type signals because of the self-similarity properties of some wavelet basis sets (Ciuciu et al., 2012). For instance, multispectral wavelet filters may be constructed by simply dilating and translated a compactly supported kernel (a wavelet function, ψ). Such continuous wavelet transforms facilitate a time-frequency signal decomposition across a continuous range of scales (Grossmann and Morlet, 1984; Kronland-Martinet et al., 1987; Billings and Keilholz, 2018). Orthonormal wavelet bases (ψ, and the scaling functions, ϕ) may also be constructed to afford a discrete segmentation, and a perfect reconstruction, of an input signal across multiple resolutions (Daubechies, 1988, 1992). Since their development, wavelets have become an important tool in fMRI analysis (Bullmore et al., 2004). Several methodological studies have shown the usefulness of combining wavelet filtering with various connectivity metrics to better characterize FC networks (Achard and Bullmore, 2007; Sato et al., 2007; Chang and Glover, 2010; Eryilmaz et al., 2011; Guo et al., 2012; Schröter et al., 2012). These and other methods have been extended into investigations of fMRI based biomarkers for neurological diseases such as addiction (Salomon et al., 2012; Lam et al., 2013), depression (Salomon et al., 2011; Meng et al., 2013), Parkinson's (Skidmore et al., 2011), Alzheimer's (Supekar et al., 2008; Wang et al., 2013), and schizophrenia (Alexander-Bloch et al., 2010; Bassett et al., 2012).

The present study seeks to characterize the BOLD signal's functional connectivity across multiple spectral scales. The study is motivated by findings from multiple sources citing patterns in FC-fMRI organization at in frequency bands within and beyond the habitually sampled low-frequency fluctuation (LFF) range (0.01–0.1 Hz). For instance, Kalcher et al. (2014) demonstrated large FC network variations among tissue types and gray-matter seed-regions when tissues and ROIs were filtered into different passbands (<0.1 Hz; 0.1–0.25 Hz; 0.25–0.75 Hz; 0.75–1.4 Hz). Wu et al. (2008) showed that cortical networks tend to organize in the frequency range between 0.01 and 0.06 Hz while limbic networks organize between 0.01 and 0.14 Hz. Chang and Glover (2010) showed that the frequency band harboring maximal correlation strength within the default mode network changed over time. Billings et al. (2017) mapped these multispectral fluctuations onto a 2-dimensional neighborhood embedding. The present study uses a series of data-driven techniques to observe how BOLD FC networks differ across a multiscale wavelet bases.

## Materials and methods

### Data acquisition

Neuroimaging data were downloaded from the 1000 Functional Connectomes Project website (Milham, 2013), specifically, the *Enhanced Rockland Sample Multiband Imaging Test-Retest Pilot Dataset* uploaded by the Nathan Kline Institute for Psychiatric Research (Nooner et al., 2012; Nathan Kline Institute for Psychiatric Research, 2013). This dataset was chosen as it was one of the first to make use of multiband imaging (Feinberg et al., 2010) to produce BOLD scans with short repetition times (*TR* = 0.645 s). Study data were derived from 32 individuals randomly chosen from the database (n. female = 22, n. right handed = 31, n. no handedness = 1, mean age = 44 y, std. age = 18 year). One volunteer's data was excluded after becoming corrupted during preprocessing.

Each volunteer's dataset consisted of whole-brain BOLD-weighted functional scans acquired on a 3T Siemens Magnetome TriTom (multiband EPI; TR 645 ms; TE 30 ms; 40 slices; FOV 22.2 cm × 22.2 cm; 3 mm isotropic voxels; 900 images). A 32-channel anterior/posterior head coil facilitated multiband EPI imaging at high temporal resolution. An MPRAGE scan was acquired to facilitate alignment (TR 1900 ms; TE 2.52 ms; 176 slices; FOV 25 cm × 25 cm; 1 mm isotropic voxels).

### Preprocessing

A series of preprocessing steps were carried out over the entire data set to bring data points into temporal and spatial alignment. These steps were conducted using revision 6,470 of the Statistical Parametric Mapping MATLAB toolbox (Friston et al., 2011). Slice timing mismatches were corrected per each slice's multiband acquisition time. Within-scan images were realigned to correct for movement between repetitions. Each scan's mean realigned image was co-registered to the volunteer's structural image. Structural images were segmented into 5 tissue classes: gray matter, white matter, cerebrospinal fluid (CSF), bone, and soft tissue. A warping matrix was evaluated and used to normalize each scan from subject space to MNI space. Images were smoothed by an 8 × 8 × 8 mm Gaussian kernel. Volunteer images were realigned to the group mean of the functional images. A gray-matter mask was applied to all images. Voxels included in the mask were required to have at least a 50% probability of containing gray matter across all volunteers. Finally, motion terms were regressed from voxel time-series.

### Multispectral decomposition, the wavelet packet transform

The wavelet packet transform (WPT) is a generalization of domain transforms utilizing orthonormal wavelet bases (Daubechies, 1988; Coifman and Wickerhauser, 1992; Coifman et al., 1992). The WPT is conducted via iterative convolutions of an input signal, *x*(*t*), with paired high-pass and low-pass filters, *h* and *g*. The filters are quadrature mirrors of one another and divide the input into orthogonal subbands. Successive filtering operations produce trees of wavelet packet coefficients over *d* ∈ [0, 1, 2, …, ∞] sets of 2^d^ evenly segmented subbands. Application of the WPT filtering schema *d* times is called the decomposition's “depth.” The set of “positions,” *p* ∈ [0, …, 2^*d*^], denote frequency ranges of packets at depth *d*. The zeroth depth is the space of the broadband signal. Each of the zeroth positions is a fully low-pass filter of variable width. The range of each packet's passband is roughly equivalent to $\left[\frac{p\;\left(\frac{f_{s}}{2}\right)}{2^{d}},\frac{\left(p+1\right)\;\left(\frac{f_{s}}{2}\right)}{2^{d}}\right]\left(Hz\right)$, where *f*_*s*_ is the sampling frequency. In the present study, the filtered data existing at depth *d*_*i*_ and position *p*_*j*_ is given the shorthand notation “D*d*_*i*_P*p*_*j*_.” Thus, the D2P0 signal is quarter-band signal covering the lowest frequencies, and the D2P3 signal is the quarter-band signal covering the highest frequencies.

For the present study, we generated a filterbank from Daubechies' 7-tap wavelet. The Daubechies family of wavelets offers the highest number of vanishing moments, or taps, for a given support width. Increasing the number of taps sharpens the filter edges in the Fourier domain at the cost of increased filter length (i.e., blurring in the time domain). Daubechies' 7-tap wavelet produces short duration filters with good spectral separation. Each voxel signal was filtered into packet coefficients at all positions of WPT depths 0 through 6, generating a total of 127 subbands. For more details on WPT theory and usage, the reader is referred to Supplemental Figure S1, the works of Coifman (Coifman et al., 1992), Daubechies (Daubechies, 1988, 1992), Mallat (Mallat, 1989, 1999), and Meyer (Meyer, 1993), as well as the technical notes of Misiti et al. (2013).

### Data structure

Reorganization of individual datasets for multi-subject hierarchical clustering was performed by concatenating the coefficients of a single wavelet packet, voxel-by-voxel, from all brain voxels, and from all volunteers, into spectrally-delimited group-level datasets.

### Hierarchical clustering (HC)

HC organizes a collection of data into distinctive groups through a deterministic algorithm. First, a distance metric, *S*1(*i, j*), is calculated between all *i* and *j* indices of voxel signals. In the present study, we followed the practice of defining functional connectivity via the Pearson correlation distance over real valued wavelet coefficients. Voxels and/or clusters of voxels are then clustered together, beginning with the closest voxels/clusters, and continuing until only a single cluster exists. After each clustering step, an updated distance metric, the linkage distance, *S*2(*a, b*), is calculated between all clusters *a* and *b*. For the present study, the linkage distance is defined as the average of the correlation distances between voxels in each cluster:

(1) $$S2\left(a,b\right)=\frac{1}{\left(n_{a}n_{b}\right)}\sum_{i=1}^{n_{a}}\sum_{j=1}^{n_{b}}S1\left(i\in a,j\in b\right).$$

Variables *n*_*a*_ and *n*_*b*_ are the number of voxels contained within clusters *a* and *b*. Further details on hierarchical clustering may be found in Supplemental Figure S2.

### FC networks clustered against dendrogram inconsistencies

An HC map's hierarchy may be visualized by plotting successive links as a dendrogram. For the dendrograms of the present study, voxels are ordered along the abscissa, and the linkage distance numbers the ordinate axis. Horizontal lines are plotted between clusters joined at a given linkage distance. Vertical lines measure the linkage distance between ‘successive clusters. Voxels are ordered along the abscissa in such a way as to minimize the length of each horizontal link. This arrangement results in the most related clusters being arranged adjacent to one another along the abscissa, i.e., the order of voxels along the abscissa is a linear projection of cluster similarity. A pictorial description of this process may be found in Supplemental Figure S2.

Concrete clusterings are produced by pruning links between intermediate clusters in the HC dendrogram. One method of dendrogram pruning identifies a threshold linkage distance that demarcates a specified number of clusters. For this study, the choice of how to prune the HC map was informed by calculating the inconsistency value of each link in the HC map. The inconsistency value of each link quantifies the relative change in linkage distance(s) between each link and up to *g* − 1 previous links. The higher the inconsistency value, the more dissimilar are the elements connected at that particular link relative to the elements connected beneath that link (Zahn, 1971). Small values for the variable *g* bring the inconsistency algorithm to focus on locally inconsistent links in the HC map. Alternatively, larger values of *g* will search the area below each link to provide a more globally representative assessments of cluster inconsistency. For a given HC map, the *k*^*th*^ link's inconsistency value is calculated as *Y*_4_(*k*) = (*z*(*k*) − *Y*_1_(*k*))/*Y*_2_(*k*). Where *Y*_1_(*k*) is the mean of the linkage distances for the *k*^th^ link and the first *g* − 1 links beneath it. The quantity *Y*_2_(*k*) is the standard deviation of the *k*^th^ set of linkage distances. The quantity *z*(*k*) is the linkage distance of the *k*^th^ link. Having set the *g-*value to perform either a local (*g* = 2) or a global search (*g* ≫ 2), we select a threshold level of inconsistency values above which to remove all of the most inconsistent links, and all of their dependents. By pruning the HC tree along natural cleavage points, natural clusterings may be better resolved.

### Quantifying FC network similarity

We utilized a mutual information-based criterion to compare parcellations of FC networks. Specifically, we use Marina Meila's normalization for mutual information between clusterings called the variation in information *(VI)* (Meilǎ, 2007):

(2) $$VI\left(C^{\prime },C^{\prime \prime }\right)=\;\left[H\left(C^{\prime }\right)-I\left(C^{\prime },C^{\prime \prime }\right)\right]+\;\left[H\left(C^{\prime \prime }\right)-I\left(C^{\prime },C^{\prime \prime }\right)\right].$$

Here, *H* is the entropy of a clustering, $H\left(C\right)=-\overset{k}{\underset{i=1}{\sum }}P\left(i\right)log_{2}P\left(i\right)$, with *P*(*i*) the probability, $\frac{\left|C_{i}\right|}{n}$, of choosing a voxel from the ith cluster in *C* from all *n* voxels. The term *I* is the mutual information between clusterings, $I\left(C^{\prime },C^{\prime \prime }\right)=\overset{k}{\underset{i=1}{\sum }}\overset{l}{\underset{j=1}{\sum }}P\left(i,j\right)log_{2}\frac{P\left(i,j\right)}{P\left(i\right)P\left(j\right)}$, where $P\left(i,j\right)=\;\frac{\left|{C^{\prime }}_{i}\cap {C^{\prime \prime }}_{j}\right|}{n}$. The first term in equation (2) may be thought of as how much information is lost when going from clustering C' to C”. The second term is then how much information is left to be gained when going from C' to C” (Wagner and Wagner, 2007).

### Voxelwise comparisons of FC networks

One important question to ask when comparing multispectral realizations of FC networks is how specific brain regions contribute to whole-brain network variability. The approach used in the present study characterized voxelwise connectivity as the degree of overlap between each voxel's nearest neighbors, as expanded between spectrally delimited FC graphs. Specifically, the Jaccard distance compared how similar the nearest 5% of correlating voxels are in each subband network:

(3) $$JD_{vw}=\frac{\#\left[\left(v_{j}\neq w_{j}\right)\cap \left(\left(v_{j}\neq 0\right)\cup \left(w_{j}\neq 0\right)\right)\right]}{\#\left[\left(v_{j}\neq 0\right)\cup \left(w_{j}\neq 0\right)\right]}.$$

The Jaccard distance quantifies the percentage of binary elements that differ between sets *v* and *w*. Results were reported as the average voxel-wise Jaccard distance across volunteers. Analysis was limited to the D6P1 (12–24 mHz), D5P1 (24–48 mHz), D4P1 (48–97 mHz), D5P4 (97–121 mHz), D5P5 (121–143 mHz), and D4P3 (141–194 mHz) packets because potentially divergent FC networks were consistently produced by packets in these ranges (see Discussion and Results). Each packet graph was compared to the graph constructed from wideband BOLD images. Wideband images were generated from the inverse WPT of only the six aforementioned packets (coefficients from other packets were set to zero before taking the inverse).

## Results

### Functional connectivity maps across spectra

**Figure 1:**
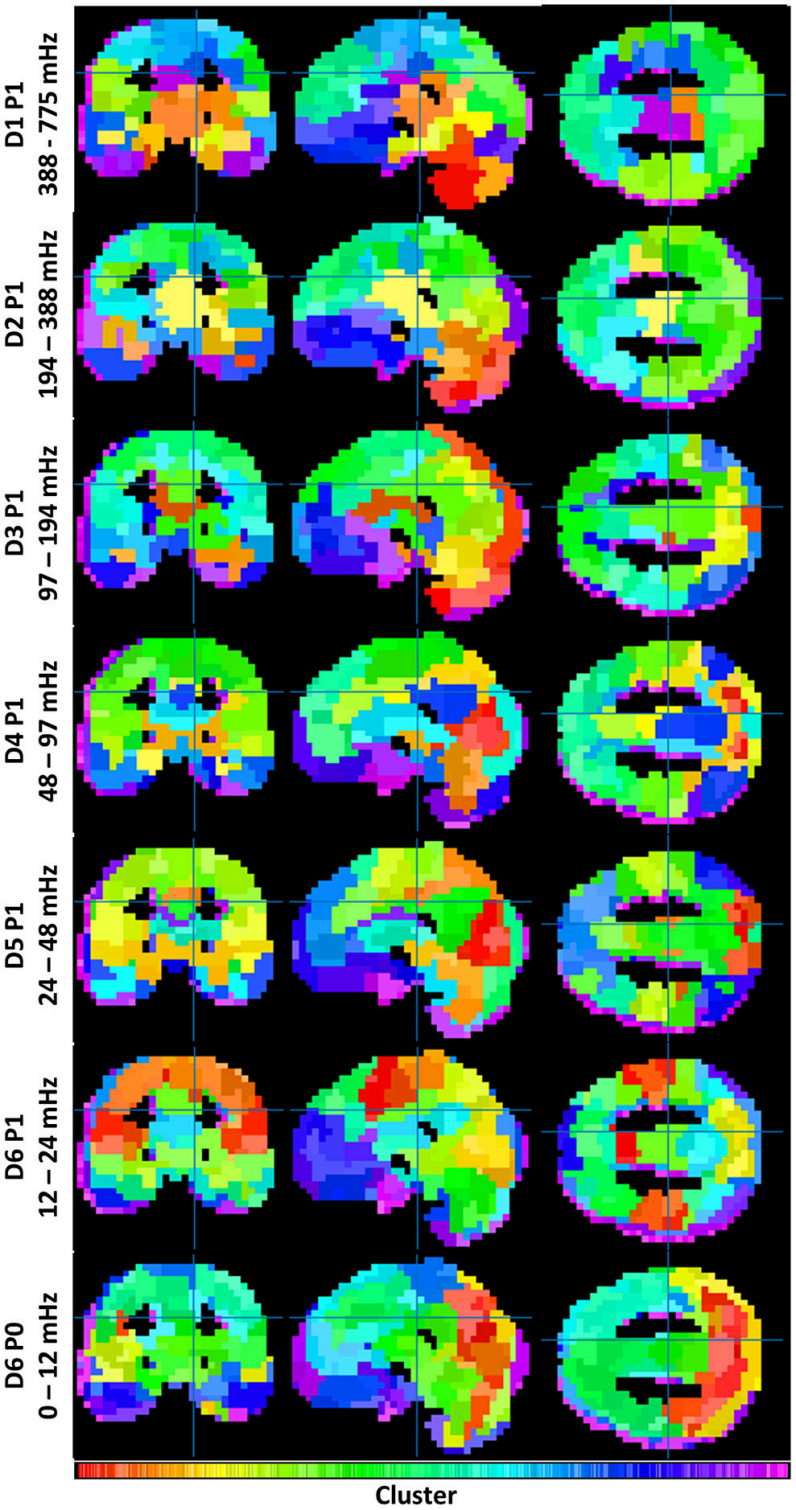
Illustrates the similarities and differences between functional connectivity networks across spectra. Each clustering contains 355 ± 4 clusters (see Supplemental Figure 3). Coloration is a projection from each cluster's location on its dendrogram onto a 1D colorbar (see Supplemental Figure 2).

To understand the overall variation of FC-fMRI networks across spectra, Figure 1 displays their cross-sectional views. Owing to space limitations, only a subset of packet networks are shown. Displayed packets follow the discrete wavelet transform schema, a multiresolution filter bank spanning the full spectral range without overlap. Each subband network was realized as a clustering with 355 ± 4 clusters. The number of clusters was derived upon consultation with the inconsistency values across packets (*g* >> 2, for a global search). These data are provided in Supplemental Figure S3.

Both similarities and differences exist in the networks produced within each subband. Whereas FC networks in the LFF range possess many of the networks expected from previous studies—including a default mode network, a somatomotor network, frontal and visual networks, etc.—such networks become less defined at frequencies above 0.2 Hz. Rather, these frequencies produce FC networks with increased segmentation among midbrain and brainstem regions, and with reduced segmentation among cortical regions. A category of mid-frequency fluctuations (MFF) (0.1 – 0.2 Hz) displays a mixture of increased midbrain/brainstem segmentation with some cortical segmentation (e.g., the bilateral angular gyri of the default mode). DC frequency information also resembles known cortical brain networks; however, the networks appear blurred by comparison to networks constructed with LFF's.

### Variation in information (VI) across spectra

**Figure 2:**
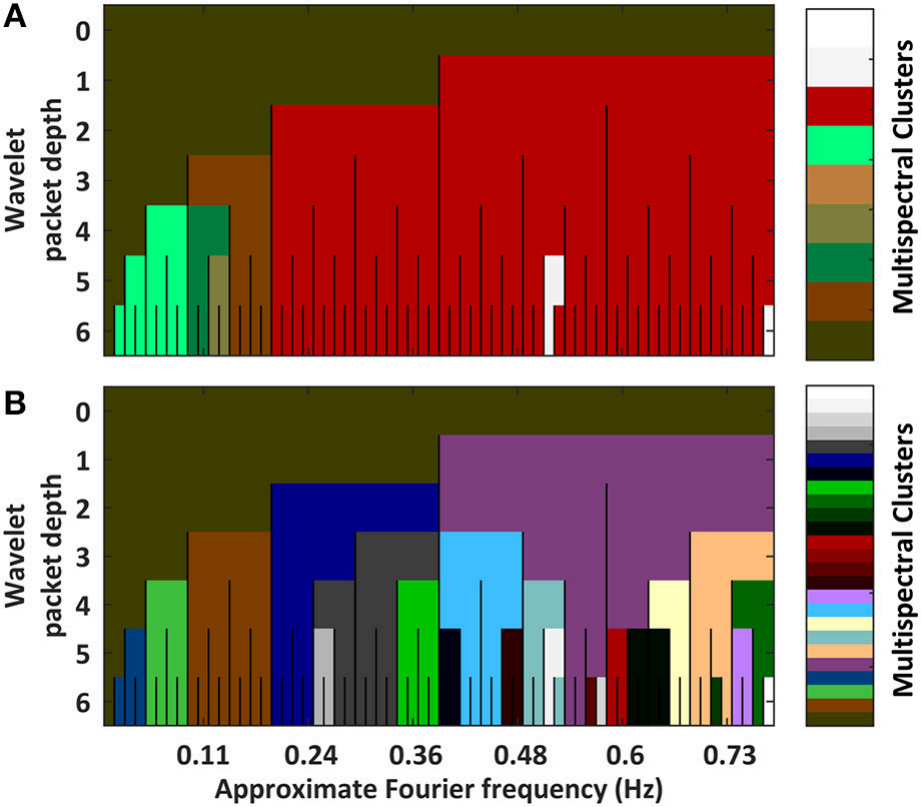
Plots hierarchical clusterings of the similarities between functional connectivity networks across spectra. The distance metric was variation in information between concrete clusterings (Intermediate results are provided in Supplemental Figures 3, 4). To better assess the decomposition's natural segmentation, the dendrogram was pruned at a coarse scale **(A)** and at a fine scale **(B)** (Associated dendrograms are displayed in Supplemental Figure 5). Overall, networks segment into passbands. Sub-bands containing DC frequencies self-associate. Granular differences among high frequency packets are likely artifactual owing to increased noise at high frequencies.

We can quantify the relatedness between spectrally delimited functional connectivity networks by assessing the *VI* between clusterings. A triangle plot of inter-spectral FC BOLD network VI distances is provided in Supplemental Figure S4. These distances are used in a hierarchical clustering (Supplemental Figure S5). Links were quantified via the “average” linkage metric. The plots in Figure 2 show the results from pruning the dendrogram in two ways. Part A of the figure shows a coarse clustering from pruning the link having the single highest local inconsistency value (g = 2). Part B of the figure shows a finer clustering that removes the first inconsistency value (g>>2) between any two packets in the LFF range.

Part A of the figure shows that the single largest jump in linkage distance occurs when connecting the D5P4 and D5P5 packets. This is an indication that sharp differences exist between FC networks above and below approximately 0.12 Hz. Alternatively, if inconsistency values are stabilized by averaging the change in linkage distances over a large number of previous links (g>>2), FC networks are shown to segment into a multiresolution filterbank of passbands (i.e., the set of wavelet packets in the first position of each depth). In both clusterings, FC networks containing DC frequencies form a separate group.

Taken as a whole, FC networks appear to segment into at least four types when drawing from different spectral components: (1) networks of 0.01 to 0.1 Hz LFF's, (2) networks of >0.2 Hz high-frequency fluctuations, (3) networks of 0.1–0.2 Hz MFFs, and (4) networks of DC frequency fluctuations. Additional varieties of FC networks may exist within finer passbands in the LFF and MFF ranges.

### Voxelwise connectivity between spectra

**Figure 3:**
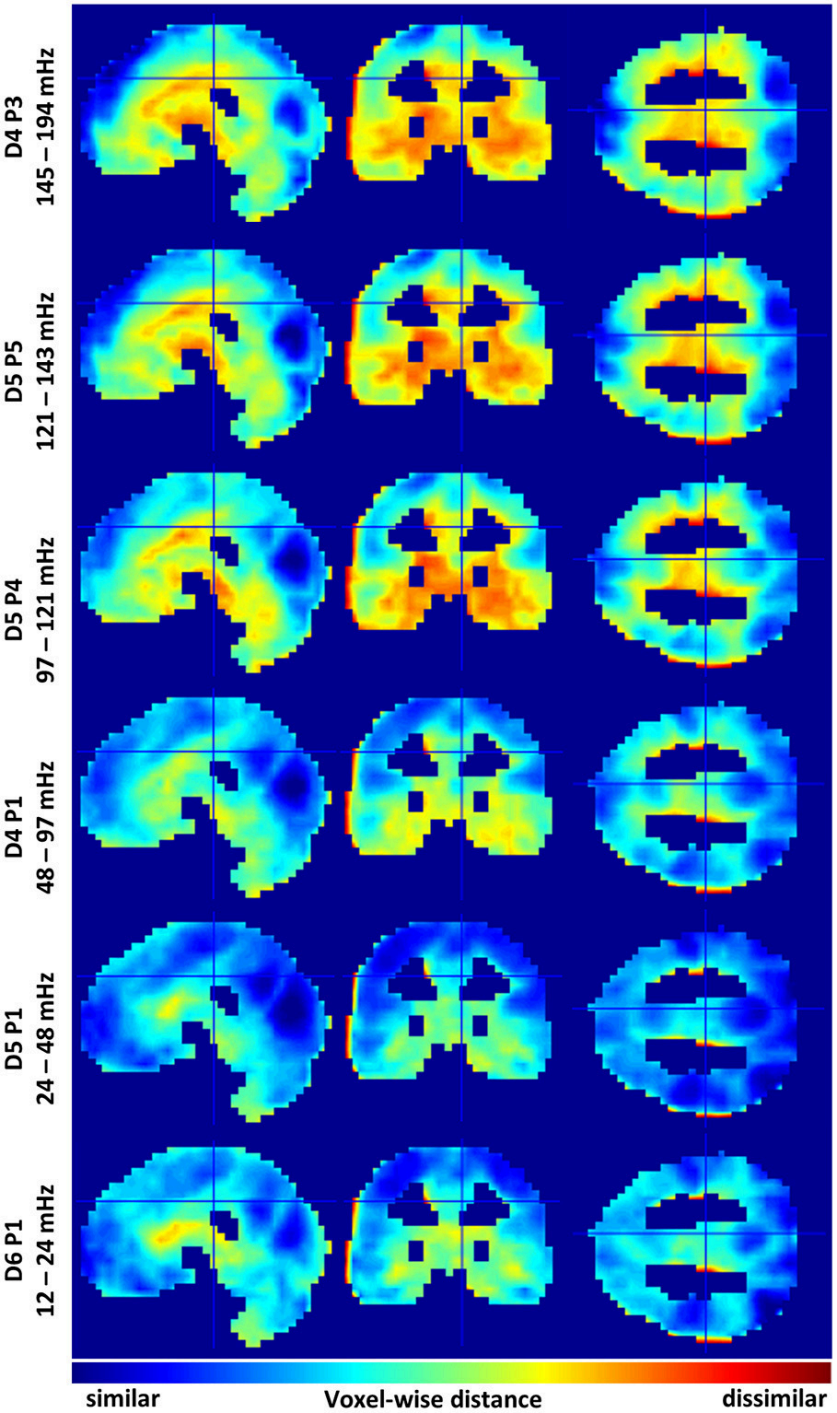
Identifies similarities and differences in voxelwise functional connectivity graphs among selected sub bands. Variations are relative to the mean across the six sub bands. Cool colors indicate voxels sharing similar functional connectivity graphs. Warm colors demarcate dis-similarly connected voxels. Data histograms are provided in Supplemental Figure 6. Images displaying data standard deviations are provided in Supplemental Figure 7. The supplemental tables provide neuroanatomical labels for the most similar and dissimilar regions.

A good way to assess differences between multispectral FC-fMRI networks is to observe differences in the group membership of individual voxels. To this end, we calculated Jaccard distances between the nearest neighbors (via correlation) of each voxel, in each spectral subband, vs. the correspond voxel from wideband filtered images. Slice representations of voxelwise network comparisons are shown in Figure 3. A series of tables detailing the 20 regions with the most similar and the least similar connectivity patterns from each subband are provided in Supplemental Tables 1–6. Histograms of the mean Jaccard distances are provided in Supplemental Figure S6. Supplemental Figure S7 displays standard deviations of Jaccard distances for reference.

Regions showing marked similarity across spectra include many areas of the cerebral cortex, including, the intracalcerine cortex, the lateral occipital cortex, the lingual gyrus, precuneous, precentral gyrus, frontal pole, and post-central gyrus. LFFs from the D5P1 packet (0.24 and 0.48 Hz) show the strongest voxelwise similarity with the spectral average (mean *JD* ≅ 0.5). Networks produced by frequencies above and below the D5P1 band show less similar voxelwise correlation in cortical regions. Additionally, these spectra show many differences in the correlation neighborhood of voxels in regions of the midbrain, basal ganglia, and the temporal lobe, including, the globus palladus, the thalamus, the hippocampus, the caudate, and the temporal pole. The most extreme deviations from the spectral average are observed from MFF packets above 0.12 Hz. The mean voxelwise Jaccard distance is ~0.8 for packets D5P5 (121–143 mHz) and D4P3 (141–194 mHz). The the mean *JD* is ~0.6 for the four other lower frequency packets (see Supplemental Figure S6).

## Discussion

It is common practice in fMRI studies to band-pass filter signals to the LFF range (Biswal et al., 1996; Murphy et al., 2013). The present study confirms the utility of this practice while providing insights into its limitations. Figure 2A Demonstrates that the connectivity structure of BOLD fluctuations can form a homogenous LFF group. But this LFF group structure is seen as homogenous only relative to a sharp change in network structure occurring at ~0.12 Hz. An alternative perspective which takes more information about the evolution of each cluster into account (Figure 2B) demonstrates that LFF networks may form two distinct networks before differences in an MFF network are observed. A look at the associate dendrogram shows that both ways to segment networks in the 0.01–0.2 Hz range may be equally valid (Supplemental Figure S6). Indeed, the LFF and MFF networks cluster together later in the dendrogram. Similar trends are observed in previous studies using images from the same volunteers but with different EPI parameter choices (Billings, 2017).

While heterogeneous network properties across spectra are often observed in electroencephalographic measurements (Lu et al., 2007; Mantini et al., 2007), the presence of multispectral network diversity in the BOLD signal is only recently beginning to emerge. Zuo et al. (2010) and Xue et al. (2014) observed differential activation patterns in slow-4 (0.027–0.073 Hz ~ D5P1) vs. slow-5 (0.01–0.027 Hz ~ D6P1) FC-fMRI activity. Similarly, Thompson and Fransson (2015) demonstrated that the centers of graph-theoretic hubs in cortical networks are frequency dependent.

Having oversampling multispectral BOLD FC clusterings, the present study selected a set of 6 passbands with potentially distinct network properties (Figure 3). From these 6 passbands, it appeared that a subband of the LFF range—the D5P1 packet network—was very similar to the wideband average. As found by Wu et al. (2008), networks in higher (MFF) frequencies tended to hold unique connectivity structures in limbic regions, e.g., the orbitofrontal cortex, hippocampus, and temporal pole. Indeed, as MFFs and high frequency fluctuations acquire increased differentiation among brain stem and midbrain regions, they appear to lose some expected connectivity structures in cortical regions (Figure 1). Notwithstanding, Boubela et al. (2013) observed prototypical resting-state networks in BOLD data sampled above 0.25 Hz. Kalcher et al. (2014) confirmed the presence of long-range functional connectivity at high frequencies from rapid TR BOLD data.

At the low end of the LFF frequency range (0.01–0.024 Hz, D6P1 packet) cortical networks were similar to the wideband average. By comparison, DC frequency networks appear blurred. The blurring is likely from a noise source as DC frequency networks structures are surprisingly similar despite the presence of any higher frequency information. Birn et al. (2013) noted that longer scans increase test-retest reliability of FC studies. Methods from the present study may be adapted to investigate if and how very slow brain rhythms (<0.01 *Hz*) coordinate unique functional networks.

The present study observed that FC networks establish the appearance of limbic MFF networks and cortical LFF networks. Hypothetically, this is an indication that slow cortical dynamics emerge from rapid information exchange among deeper brain structures. If this is the case, then the difference maps in Figure 3 may show the accumulation of rapid (>0.12 Hz) limbic activity into slow (0.024 and 0.048 Hz) cortical structures. Alternatively, MFF BOLD signaling could be a kind of structured noise.

The presence of noise confounds is the chief concern limiting the interpretation of study results. The gray-matter mask of the present study included any voxel having at least a 50% probability of containing gray matter in all volunteer images. Some voxels were thereby included from outside gray matter (e.g., from cerebrospinal fluid, white matter, and extra-cerebral tissues). For instance, in Figures 1, 3, voxels at the edges of gray matter regions appear to segment into their own clusters. Some anatomical locations labeled in the supplemental tables mark points in these clusters. Better segmentation of gray matter regions may remove these confounds. None-the-less, the smooth transition from limbic to cortical network types as brain rhythms slow was observed in pairwise correlations between very many gray matter voxels.

Observations of multispectral variability in brain FC are contrary to the expectation that 1/f-type systems are scale-free. There are, however, other interpretations that admit to the simultaneous presence of 1/f-type power spectral densities alongside unique multiscale structures. Namely, unique large-scale structures may be emergent properties of multiscale granular activities. In the case of the brain, very many binary action potentials must somehow sum to become a lifetime of thoughts and feelings. Theoretically, the capacity for a system to share information across scales is a measure of the system's complexity (Wolfram, 2002). Natural complex systems like the brain must simultaneously build large-scale structures from granular processes and fine-tune multiscale functions with subband information. The unique information bearing capacity of both granular and coarse measures of natural complex systems should therefore encourage FC-fMRI studies to leverage multispectral basis transforms (Billings and Keilholz, 2018).

## Data availability statement

The original data supporting the results of this article are freely available from http://fcon_1000.projects.nitrc.org/indi/pro/eNKI_RS_TRT/FrontPage.html

## Author contributions

All authors designed the study. JB conducted the analysis with advice from all authors, especially AM and SK. JB authored the manuscript with input and revisions provided by all authors. Each author has given final approval of the manuscript's publication and agrees to be accountable for all aspects of the work.

### Conflict of interest statement

The authors declare that the research was conducted in the absence of any commercial or financial relationships that could be construed as a potential conflict of interest.

## Abbreviations

FC: functional connectivity
fMRI: functional magnetic resonance imaging
FC-fMRI: functional connectivity of functional magnetic resonance imaging data
*TR*: repetition time
LFF: low-frequency fluctuations (0.01–0.1 Hz)
MFF: mid-frequency fluctuations (0.1–0.2 Hz)
WPT: wavelet packet transform
D*i*P*j*: indices for the wavelet decomposition depth (D) *i* and position (P) *j*
HC: hierarchical clustering
VI: variation in information.
