# Supplementary material for: Disentangling Multispectral Functional Connectivity With Wavelets
Source: Front Neurosci. 2018 Nov 6;12:812. doi: 10.3389/fnins.2018.00812 (PMC6232345; doi:10.3389/fnins.2018.00812)
Supplement: Supplementary file 1 [file Table_1.PDF]

Title Supplemental Table 1: Group average Jaccard distance, D4P3

Thresholding 20 outermost regions from each tail of the distribution (see supplemental figure 6).

Notes Table shows all local maxima separated by more than 20 mm. Regions were automatically labeled using the HarvardOxford-maxprob-thr0 atlas. x, y, and z =Montreal Neurological Institute (MNI) coordinates in the left-right, anterior-posterior, and inferior-superior dimensions, respectively.
Data were masked with the SPM Anatomy Toolbox Atlas.
Many regions with large Jaccard distances represent noise from realignment and susceptibility artifacts. Any having anatomical labels have been struck-out to avoid confusion.

Contrast Name			MNI Coordinates		
	Region Label	Intensity	x	y	z
Positive	Left Cerebral White Matter	0.963	-17	-12	0
	Right Pallidum	0.962	18	-7	0
	Location not in atlas	0.949	-42	-7	70
	~~Occipital Pole~~	0.942	3	-107	5
	Left Cerebral White Matter	0.936	-17	-42	15
	Location not in atlas	0.932	-57	3	50
	~~Angular Gyrus~~	0.930	-57	-52	55
	Location not in atlas	0.929	-52	-32	65
	Right Cerebral White Matter	0.927	28	-2	20
	Right Cerebral White Matter	0.921	38	-27	-10
	Left Hippocampus	0.920	-27	-22	-15
	Left Lateral Ventrical	0.918	-7	3	10
	Left Cerebral White Matter	0.916	-37	-12	25
	Parahippocampal Gyrus, anterior division	0.915	-27	3	-15
	Left Cerebral White Matter	0.914	-27	13	20
	Location not in atlas	0.914	-52	-77	-25
	Temporal Pole	0.914	48	13	-25
	Right Cerebral White Matter	0.913	23	18	5
	Location not in atlas	0.913	-47	-77	45
	~~Middle Frontal Gyrus~~	0.911	-32	18	65
Negative	Lateral Occipital Cortex, superior division	0.711	28	-82	25
	Frontal Pole	0.713	13	68	15
	Frontal Pole	0.714	13	48	45
	Left Cerebral White Matter	0.714	-22	-87	15
	Occipital Pole	0.716	-27	-102	0
	Location not in atlas	0.736	-17	-92	-35
	Superior Frontal Gyrus	0.736	13	28	60
	Occipital Pole	0.736	23	-102	5
	Frontal Pole	0.736	-12	63	20
	Superior Frontal Gyrus	0.741	-17	-7	70
	Superior Frontal Gyrus	0.744	-12	28	60
	Lateral Occipital Cortex, superior division	0.745	18	-72	45
	Frontal Pole	0.745	-27	63	5
	Frontal Pole	0.747	-12	48	40
	Precentral Gyrus	0.750	-22	-27	70
	Location not in atlas	0.750	18	-87	-40
	Precentral Gyrus	0.753	28	-7	65
	Lateral Occipital Cortex, superior division	0.756	-22	-62	55
	Frontal Pole	0.756	8	63	-10
	Lateral Occipital Cortex, superior division	0.764	-47	-82	20

Title Supplemental Table 2: Group average Jaccard distance, D5P5

Thresholding 20 outermost regions from each tail of the distribution (see supplemental figure 6).

Notes Table shows all local maxima separated by more than 20 mm. Regions were automatically labeled using the HarvardOxford-maxprob-thr0 atlas. x, y, and z =Montreal Neurological Institute (MNI) coordinates in the left-right, anterior-posterior, and inferior-superior dimensions, respectively.
Data were masked with the SPM Anatomy Toolbox Atlas.
Many regions with large Jaccard distances represent noise from realignment and susceptibility artifacts. Any having anatomical labels have been struck-out to avoid confusion.

Contrast Name			MNI Coordinates		
	Region Label	Intensity	x	y	z
Positive	Right Pallidum	0.943	18	-7	0
	Left Cerebral White Matter	0.938	-17	-12	0
	Location not in atlas	0.913	-42	-7	70
	Left Cerebral White Matter	0.905	-17	-42	15
	~~Angular Gyrus~~	0.904	-57	-52	55
	~~Occipital Pole~~	0.903	3	-107	5
	Right Cerebral White Matter	0.898	28	-2	20
	Location not in atlas	0.897	-57	3	50
	Left Lateral Ventrical	0.895	-7	3	10
	Left Hippocampus	0.888	-27	-22	-15
	Right Cerebral White Matter	0.887	38	-27	-10
	Left Cerebral White Matter	0.887	-27	13	20
	Left Cerebral White Matter	0.887	-37	-12	25
	Parahippocampal Gyrus, anterior division	0.883	-27	3	-15
	~~Postcentral Gyrus~~	0.882	-42	-37	70
	Right Cerebral White Matter	0.880	23	23	-10
	Right Cerebral White Matter	0.879	38	-32	25
	Location not in atlas	0.879	-27	-37	-30
	Right Cerebral White Matter	0.877	23	-22	15
	Temporal Pole	0.877	48	13	-25
Negative	Lateral Occipital Cortex, superior division	0.616	23	-87	25
	Left Cerebral White Matter	0.622	-22	-87	15
	Intracalcarine Cortex	0.651	8	-82	10
	Frontal Pole	0.655	13	48	45
	Frontal Pole	0.667	13	68	15
	Superior Frontal Gyrus	0.671	-22	-7	70
	Superior Frontal Gyrus	0.671	-12	28	60
	Superior Frontal Gyrus	0.675	13	28	60
	Occipital Fusiform Gyrus	0.675	28	-72	-10
	Occipital Pole	0.675	-27	-102	0
	Location not in atlas	0.678	-12	-92	-30
	Frontal Pole	0.682	-12	63	25
	Occipital Fusiform Gyrus	0.685	-22	-77	-10
	Frontal Pole	0.690	-17	43	40
	Precentral Gyrus	0.691	28	-7	65
	Location not in atlas	0.691	13	-92	-30
	Superior Parietal Lobule	0.695	-27	-42	65
	Lateral Occipital Cortex, superior division	0.697	-47	-77	40
	Occipital Pole	0.701	23	-102	5
	Location not in atlas	0.707	-42	-77	-30

Title Supplemental Table 3: Group average Jaccard distance, D5P4

Thresholding 20 outermost regions from each tail of the distribution (see supplemental figure 6).

Notes Table shows all local maxima separated by more than 20 mm. Regions were automatically labeled using the HarvardOxford-maxprob-thr0 atlas. x, y, and z =Montreal Neurological Institute (MNI) coordinates in the left-right, anterior-posterior, and inferior-superior dimensions, respectively.
Data were masked with the SPM Anatomy Toolbox Atlas.
Many regions with large Jaccard distances represent noise from realignment and susceptibility artifacts. Any having anatomical labels have been struck-out to avoid confusion.

Contrast Name			MNI Coordinates		
	Region Label	Intensity	x	y	z
Positive	Left Cerebral White Matter	0.849	-17	-12	0
	Right Pallidum	0.836	18	-7	0
	Location not in atlas	0.810	-57	18	40
	Location not in atlas	0.802	-42	-7	70
	Left Thalamus	0.802	-17	-32	5
	~~Occipital Pole~~	0.799	3	-107	5
	Right Hippocampus	0.786	33	-27	-10
	Right Hippocampus	0.784	23	-37	5
	~~Angular Gyrus~~	0.782	-57	-52	55
	Left Cerebral White Matter	0.781	-42	-7	25
	Right Cerebral White Matter	0.780	28	-2	20
	Location not in atlas	0.778	-52	-27	65
	Left Cerebral White Matter	0.777	-47	-22	-15
	Frontal Orbital Cortex	0.772	28	23	-20
	Left Caudate	0.770	-12	3	15
	Left Hippocampus	0.769	-17	-17	-20
	~~Lateral Occipital Cortex, superior division~~	0.765	-37	-67	60
	Location not in atlas	0.765	-27	-37	-30
	Location not in atlas	0.763	-37	-57	-55
	Temporal Pole	0.762	-52	13	-15
Negative	Lateral Occipital Cortex, superior division	0.440	28	-82	25
	Left Cerebral White Matter	0.455	-22	-87	15
	Intracalcarine Cortex	0.472	8	-82	10
	Frontal Pole	0.488	13	48	45
	Left Cerebral White Matter	0.502	-12	-77	0
	Occipital Fusiform Gyrus	0.503	28	-72	-10
	Superior Frontal Gyrus	0.519	-12	28	60
	Superior Frontal Gyrus	0.522	-17	-7	70
	Postcentral Gyrus	0.534	-22	-42	70
	Lateral Occipital Cortex, superior division	0.536	-47	-67	35
	Lateral Occipital Cortex, superior division	0.537	48	-62	30
	Frontal Pole	0.544	-17	48	40
	Frontal Pole	0.545	-7	58	10
	Occipital Pole	0.552	13	-92	-20
	Precuneous Cortex	0.553	3	-62	30
	Location not in atlas	0.553	-12	-92	-30
	Temporal Occipital Fusiform Cortex	0.554	-27	-57	-10
	Lateral Occipital Cortex, superior division	0.556	18	-67	50
	Postcentral Gyrus	0.557	28	-37	65
	Superior Frontal Gyrus	0.559	18	-7	70

Title Supplemental Table 4: Group average Jaccard distance, D4P1

Thresholding 20 outermost regions from each tail of the distribution (see supplemental figure 6).

Notes Table shows all local maxima separated by more than 20 mm. Regions were automatically labeled using the HarvardOxford-maxprob-thr0 atlas. x, y, and z =Montreal Neurological Institute (MNI) coordinates in the left-right, anterior-posterior, and inferior-superior dimensions, respectively.
Data were masked with the SPM Anatomy Toolbox Atlas.
Many regions with large Jaccard distances represent noise from realignment and susceptibility artifacts.
Any having anatomical labels have been struck-out to avoid confusion.

Contrast Name			MNI Coordinates		
	Region Label	Intensity	x	y	z
Positive	Right Pallidum	0.783	18	-7	0
	Left Cerebral White Matter	0.762	-17	-12	0
	Location not in atlas	0.748	-57	18	40
	~~Occipital Pole~~	0.720	3	-107	5
	Location not in atlas	0.711	-42	-7	70
	~~Angular Gyrus~~	0.707	-57	-52	55
	Left Cerebral White Matter	0.694	-17	-42	15
	Right Cerebral White Matter	0.687	28	-2	20
	Frontal Orbital Cortex	0.671	-17	8	-30
	Left Cerebral White Matter	0.671	-47	-12	-15
	Parahippocampal Gyrus, anterior division	0.669	23	-22	-25
	Location not in atlas	0.669	-52	-77	-25
	Location not in atlas	0.666	-52	-32	65
	~~Lateral Occipital Cortex, superior division~~	0.666	-37	-67	60
	Left Cerebral White Matter	0.664	-27	13	20
	Location not in atlas	0.659	-52	-72	45
	Temporal Pole	0.657	38	13	-20
	Temporal Fusiform Cortex, posterior division	0.656	-32	-27	-25
	Right Cerebral White Matter	0.655	38	-32	-15
	Parahippocampal Gyrus, anterior division	0.650	18	3	-20
Negative	Intracalcarine Cortex	0.339	8	-82	10
	Lateral Occipital Cortex, superior division	0.352	28	-82	25
	Lateral Occipital Cortex, superior division	0.358	-22	-87	20
	Lingual Gyrus	0.378	18	-72	-5
	Lingual Gyrus	0.393	-12	-77	-10
	Lateral Occipital Cortex, superior division	0.398	-47	-67	35
	Precuneous Cortex	0.405	3	-57	30
	Precentral Gyrus	0.405	23	-17	70
	Frontal Pole	0.406	-2	58	15
	Lateral Occipital Cortex, superior division	0.406	48	-62	30
	Postcentral Gyrus	0.408	-32	-27	65
	Left Cerebral White Matter	0.408	-37	-77	-5
	Frontal Pole	0.412	13	48	45
	Frontal Pole	0.412	-12	48	40
	Precentral Gyrus	0.413	-57	-2	35
	Postcentral Gyrus	0.416	-17	-47	70
	Right Cerebral White Matter	0.422	58	-7	15
	Lingual Gyrus	0.422	-17	-52	-5
	Precentral Gyrus	0.424	3	-17	60
	Postcentral Gyrus	0.425	53	-12	45

Title Supplemental Table 5: Group average Jaccard distance, D5P1

Thresholding 20 outermost regions from each tail of the distribution (see supplemental figure 6).

Notes Table shows all local maxima separated by more than 20 mm. Regions were automatically labeled using the HarvardOxford-maxprob-thr0 atlas. x, y, and z =Montreal Neurological Institute (MNI) coordinates in the left-right, anterior-posterior, and inferior-superior dimensions, respectively.
Data were masked with the SPM Anatomy Toolbox Atlas.
Many regions with large Jaccard distances represent noise from realignment and susceptibility artifacts. Any having anatomical labels have been struck-out to avoid confusion.

Contrast Name			MNI Coordinates		
	Region Label	Intensity	x	y	z
Positive	Left Cerebral White Matter	0.812	-17	-12	0
	Right Pallidum	0.797	18	-7	0
	~~Occipital Pole~~	0.746	3	-107	10
	Left Cerebral White Matter	0.719	-17	-42	15
	Location not in atlas	0.692	-52	-77	-25
	Location not in atlas	0.691	-32	8	70
	Left Cerebral White Matter	0.690	-27	13	20
	Location not in atlas	0.687	-57	18	40
	~~Angular Gyrus~~	0.679	-57	-52	55
	Right Cerebral White Matter	0.674	28	-2	20
	Location not in atlas	0.666	-37	-17	75
	Location not in atlas	0.665	-52	-32	65
	Left Lateral Ventrical	0.658	-2	3	10
	Location not in atlas	0.644	-52	-72	45
	Location not in atlas	0.644	3	-97	30
	Parahippocampal Gyrus, posterior division	0.642	-17	-27	-20
	Left Cerebral White Matter	0.642	-42	-12	-20
	Right Hippocampus	0.640	38	-22	-15
	~~Precuneous Cortex~~	0.635	3	-82	50
	Frontal Orbital Cortex	0.631	28	13	-25
Negative	Occipital Pole	0.331	-17	-92	25
	Right Cerebral White Matter	0.341	23	-82	25
	Lingual Gyrus	0.366	13	-72	-5
	Left Cerebral White Matter	0.366	-12	-77	0
	Lateral Occipital Cortex, superior division	0.376	-47	-72	40
	Precuneous Cortex	0.383	3	-62	30
	Postcentral Gyrus	0.394	-47	-22	45
	Right Cerebral White Matter	0.398	58	-12	25
	Lateral Occipital Cortex, superior division	0.400	53	-62	35
	Right Cerebral White Matter	0.402	38	-77	0
	Frontal Pole	0.402	-7	58	5
	Occipital Pole	0.402	-27	-102	0
	Precentral Gyrus	0.404	-57	-2	30
	Location not in atlas	0.412	-12	-92	-35
	Precentral Gyrus	0.416	3	-17	55
	Left Cerebral White Matter	0.419	-12	53	25
	Supramarginal Gyrus, posterior division	0.420	58	-37	35
	Lateral Occipital Cortex, superior division	0.422	-22	-67	45
	Precentral Gyrus	0.424	-22	-22	65
	Precentral Gyrus	0.425	43	-17	60

Title Supplemental Table 6: Group average Jaccard distance, D6P1

Thresholding 20 outermost regions from each tail of the distribution (see supplemental figure 6).

Notes Table shows all local maxima separated by more than 20 mm. Regions were automatically labeled using the HarvardOxford-maxprob-thr0 atlas. x, y, and z =Montreal Neurological Institute (MNI) coordinates in the left-right, anterior-posterior, and inferior-superior dimensions, respectively.
Data were masked with the SPM Anatomy Toolbox Atlas.
Many regions with large Jaccard distances represent noise from realignment and susceptibility artifacts. Any having anatomical labels have been struck-out to avoid confusion.

Contrast Name			MNI Coordinates		
	Region Label	Intensity	x	y	z
Positive	Left Cerebral White Matter	0.867	-17	-12	0
	Right Pallidum	0.859	18	-7	0
	Left Cerebral White Matter	0.831	-17	-42	15
	~~Occipital Pole~~	0.817	3	-107	10
	Location not in atlas	0.794	-32	8	70
	Location not in atlas	0.790	-57	18	40
	Right Cerebral White Matter	0.787	18	-42	10
	Location not in atlas	0.785	-52	-77	-25
	Left Cerebral White Matter	0.777	-27	13	20
	Location not in atlas	0.775	3	-97	30
	~~Precuneous Cortex~~	0.764	3	-82	50
	~~Angular Gyrus~~	0.763	-57	-52	55
	Left Cerebral White Matter	0.763	-2	-27	15
	Location not in atlas	0.763	-52	-32	65
	Right Cerebral White Matter	0.759	38	-32	-10
	Right Lateral Ventricle	0.759	3	13	5
	Right Cerebral White Matter	0.758	28	-2	20
	Left Cerebral White Matter	0.755	-42	-32	-10
	Left Cerebral White Matter	0.754	-17	-2	30
	Right Cerebral White Matter	0.744	38	-12	-15
Negative	Right Cerebral White Matter	0.501	23	-82	25
	Lateral Occipital Cortex, superior division	0.513	-47	-72	40
	Left Cerebral White Matter	0.514	-17	-87	20
	Occipital Pole	0.528	-27	-102	0
	Lateral Occipital Cortex, superior division	0.531	53	-67	35
	Occipital Pole	0.537	-42	-92	10
	Intracalcarine Cortex	0.538	8	-77	5
	Location not in atlas	0.539	-22	-92	-35
	Postcentral Gyrus	0.542	-42	-22	45
	Occipital Pole	0.547	33	-97	0
	Occipital Fusiform Gyrus	0.548	-17	-77	-10
	Precuneous Cortex	0.548	-2	-57	30
	Postcentral Gyrus	0.553	53	-22	55
	Left Cerebral White Matter	0.555	-12	58	10
	Postcentral Gyrus	0.556	-32	-27	70
	Frontal Pole	0.558	8	63	-10
	Frontal Pole	0.559	8	68	15
	Postcentral Gyrus	0.561	33	-27	65
	Location not in atlas	0.566	18	-92	-35
	Lingual Gyrus	0.568	23	-62	-5
