# Supplementary figures and images for: Disentangling Multispectral Functional Connectivity With Wavelets
Source: Front Neurosci. 2018 Nov 6;12:812. doi: 10.3389/fnins.2018.00812 (PMC6232345; doi:10.3389/fnins.2018.00812)

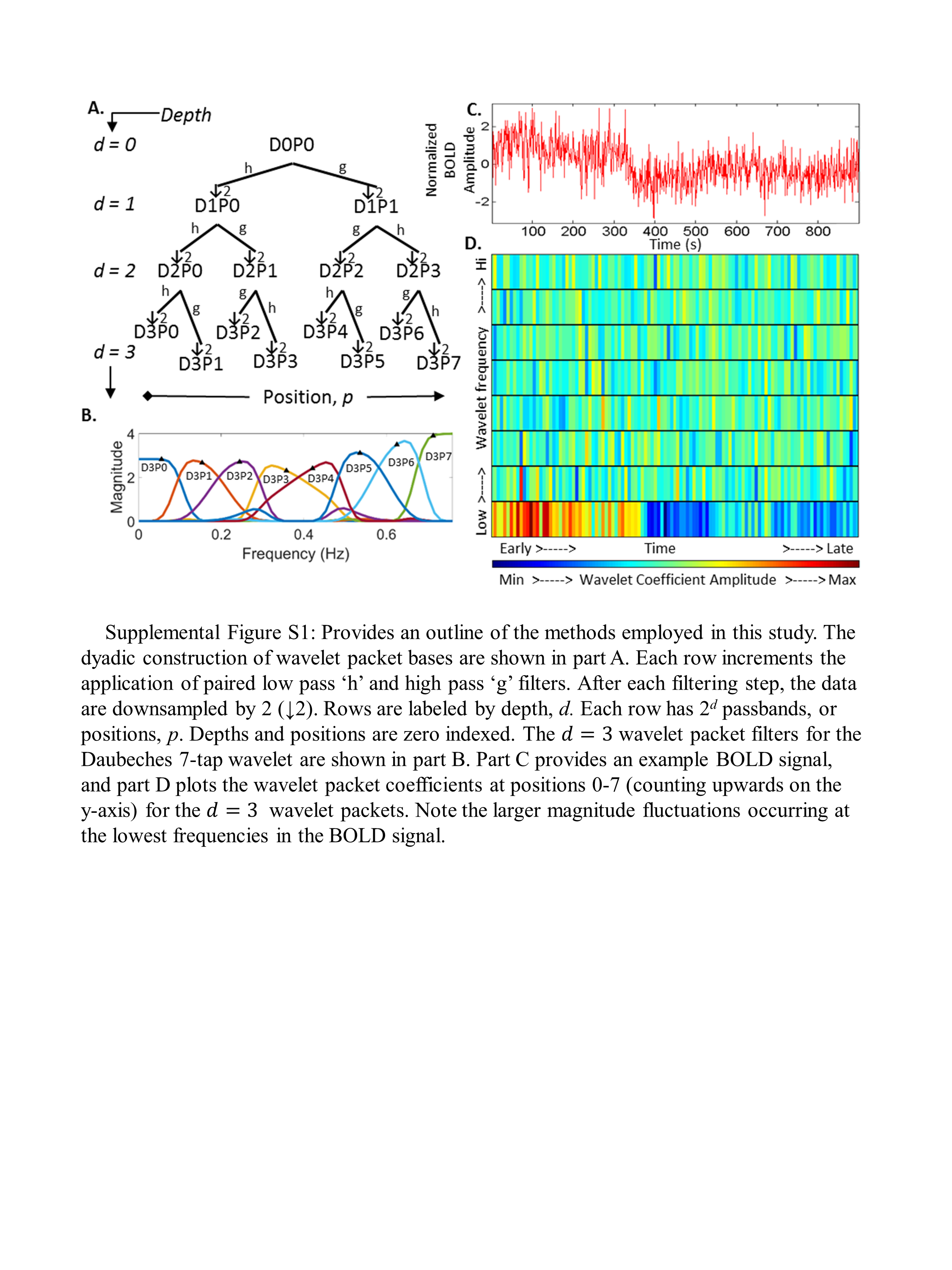

Supplement: Supplementary file 2 [file Image_1.TIF]

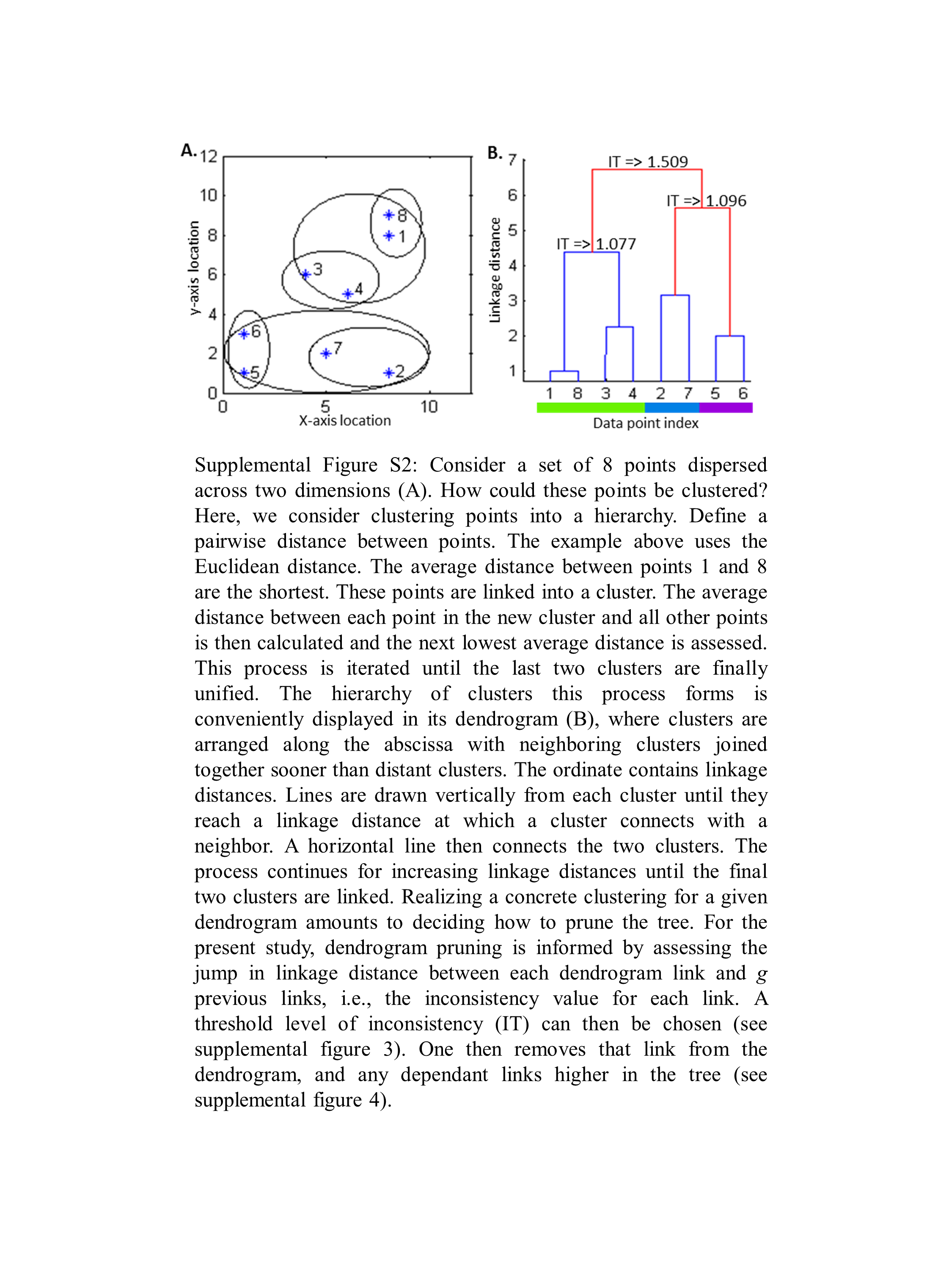

Supplement: Supplementary file 3 [file Image_2.TIF]

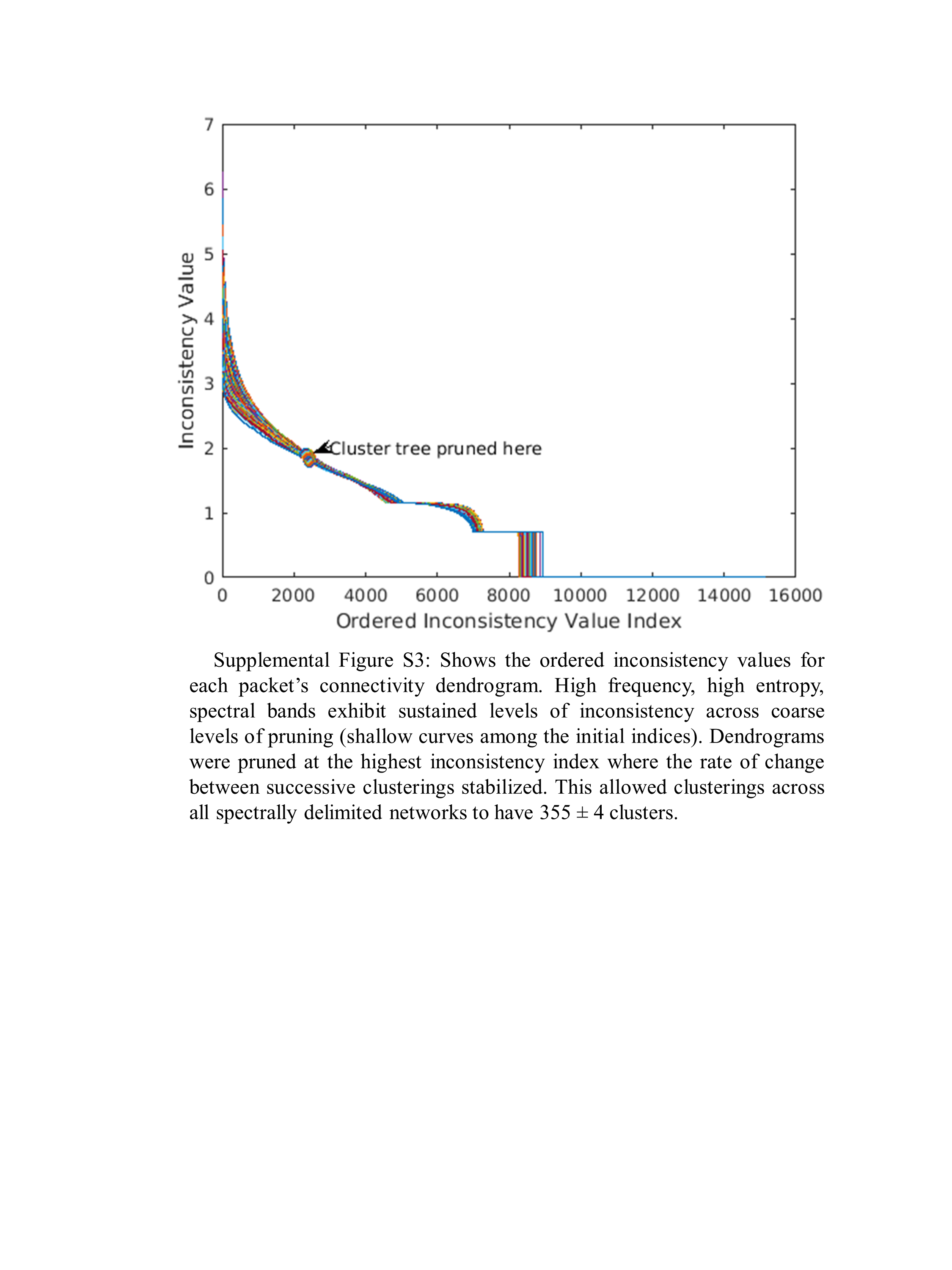

Supplement: Supplementary file 4 [file Image_3.TIF]

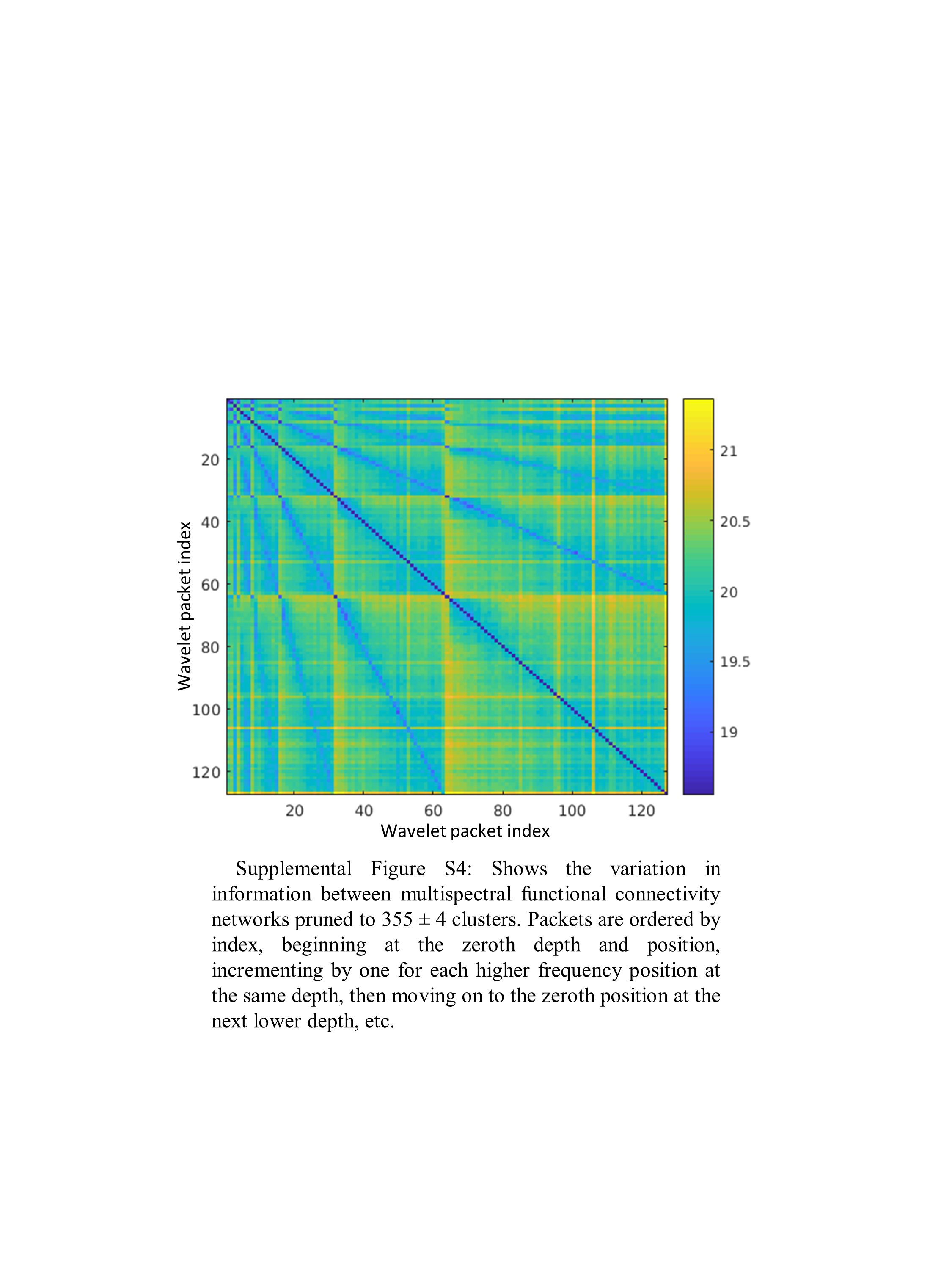

Supplement: Supplementary file 5 [file Image_4.TIF]

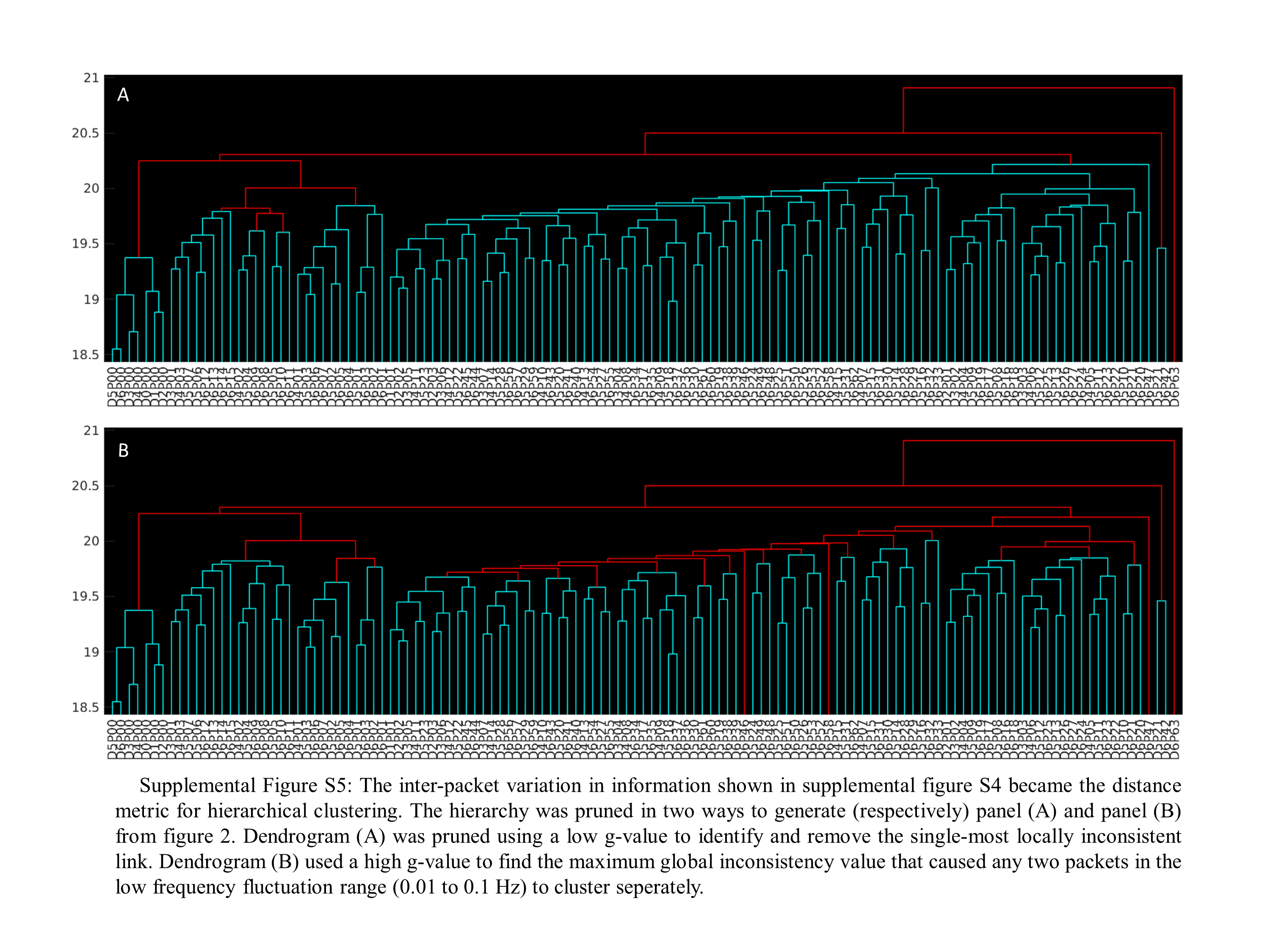

Supplement: Supplementary file 6 [file Image_5.TIF]

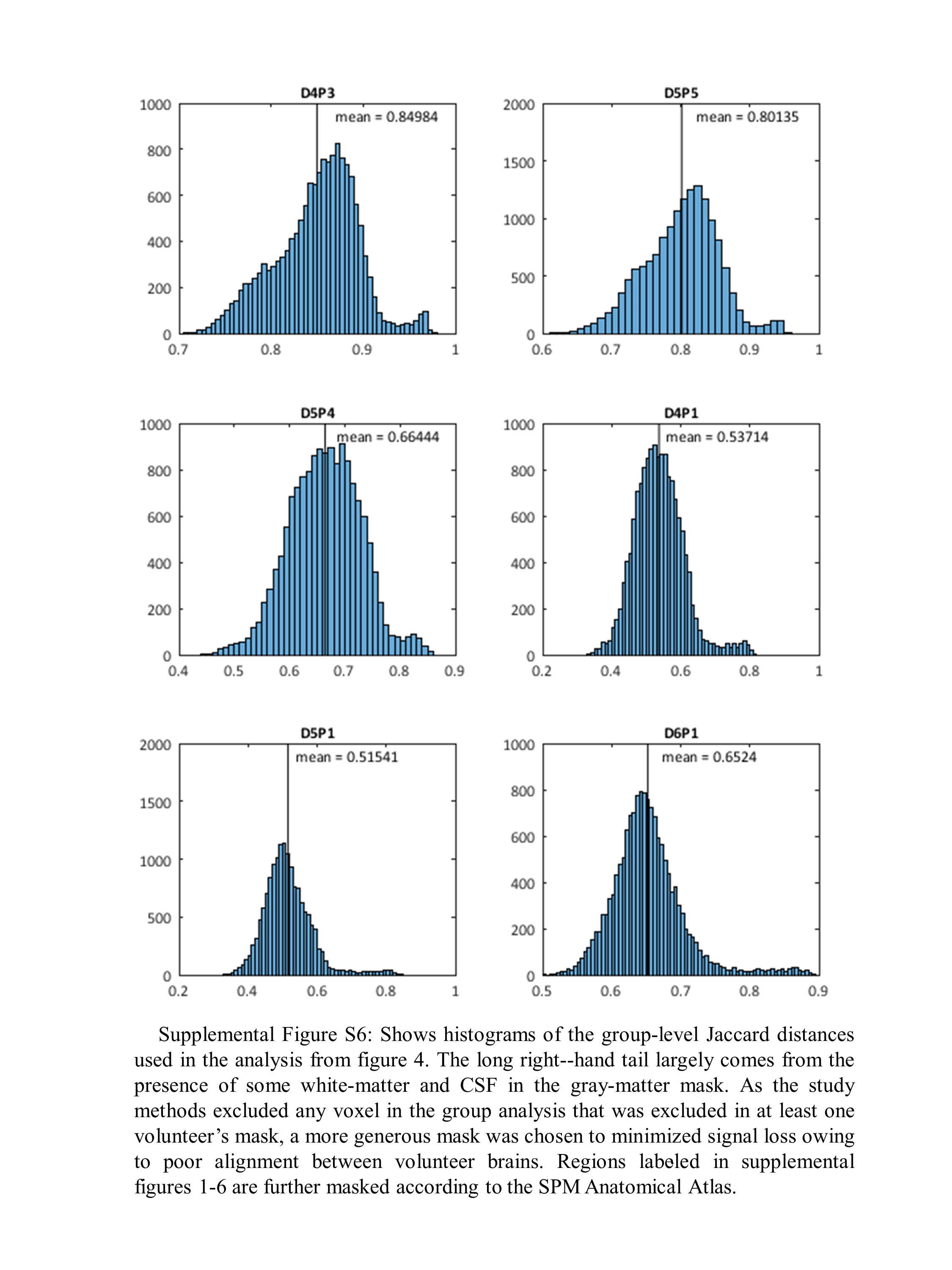

Supplement: Supplementary file 7 [file Image_6.TIF]

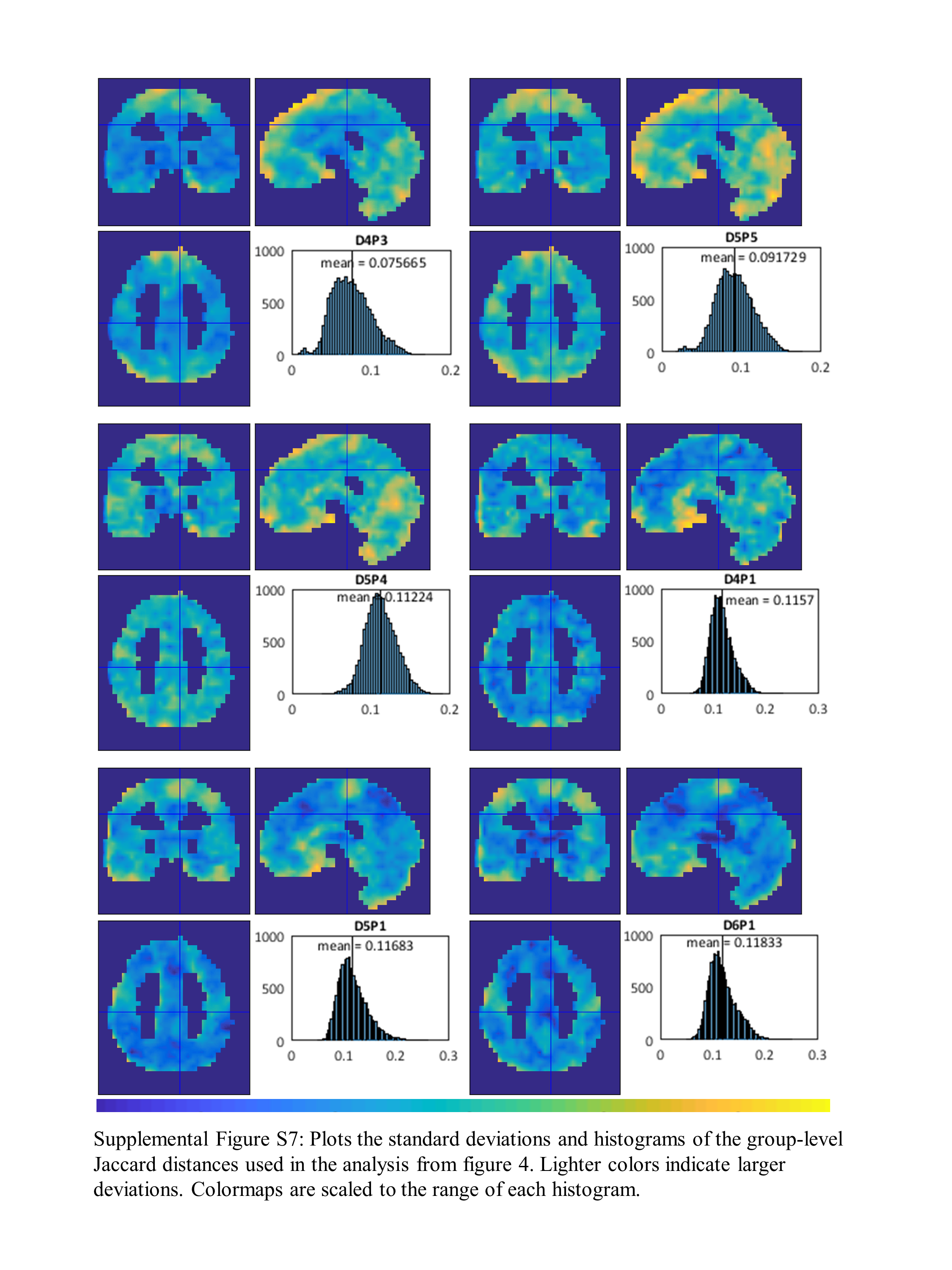

Supplement: Supplementary file 8 [file Image_7.TIF]
